# An appraisal of studies using mouse models to assist the biomarker discovery for sepsis prognosis

**DOI:** 10.1016/j.heliyon.2023.e17770

**Published:** 2023-06-28

**Authors:** Yaqing Jiao, Cindy See Wai Tong, Timothy H. Rainer

**Affiliations:** Department of Emergency Medicine, School of Clinical Medicine, The University of Hong Kong, China

**Keywords:** Mice, Prognosis, Prognostic biomarker, Sepsis

## Abstract

**Introduction:**

Clinicians need reliable outcome predictors to improve the prognosis of septic patients. Mouse models are widely used in sepsis research. We aimed to review how mouse models were used to search for novel prognostic biomarkers of sepsis in order to optimize their use for future biomarker discovery.

**Methods:**

We searched PubMed from 2012 to July 2022 using “((sepsis) AND (mice)) AND ((prognosis) OR (prognostic biomarker))”.

**Results:**

A total of 412 publications were retrieved. We selected those studies in which mouse sepsis was used to demonstrate prognostic potential of biomarker candidates and/or assist the subsequent evaluation in human sepsis for further appraisal. The most frequent models were lipopolysaccharide (LPS) injection and caecal ligation and puncture (CLP) using young male mice. Discovery technologies applied on mice include setting survival and nonsurvivable groups, detecting changes of biomarker levels and measuring physiological parameters during sepsis. None of the biomarkers achieved sufficient clinical performance for clinical use.

**Conclusions:**

The number of studies and strategies using mouse models to discover prognostic biomarkers of sepsis are limited. Current mouse models need to be further optimized to better conform to human sepsis. Current biomarker platforms do not achieve predictive performance for clinical use.

## 1 Introduction

Sepsis affects about 49 million people worldwide every year of which 20 million make it to hospital and 25% die [1]. It accounts for over 50% of all hospital deaths many of which are preventable [1]. Most patients who die from coronavirus disease 2019 (COVID-19)-associated deaths in the current pandemic would have passed through a final phase of sepsis [2]. Sepsis is a life-threatening failure of body organs caused by a dysregulated host response to infection [3]. In general, the progression involves three phases: an early regulated inflammatory phase, an intermediate dysregulated proinflammatory phase and a late dysregulated hypo-inflammatory phase in which the renal and respiratory systems are mostly implicated [3].

Although sepsis is the leading cause of death in non-coronary, critically ill patients, the identification of patients at high risk remains a challenge [4]. Many biomarkers have been evaluated for sepsis prognosis in clinical studies, including cell and receptor biomarkers, cytokine/chemokine biomarkers, acute-phase protein biomarkers, vascular endothelial damage biomarkers and coagulation biomarkers [5]. However, none of these have achieved a satisfactory specificity and sensitivity for clinical use [6]. The prediction of patient outcomes in sepsis continues to be driven by clinical signs due to the unsatisfactory performance of these currently available biomarkers [7]. The use of reliable outcome predictors would enable clinical practitioners to decide the location of care for patients, monitor the response to interventions and enrol patients in clinical trials [4]. Therefore, it is important to search for novel, effective biomarkers to better diagnose and predict sepsis progression in order to improve the outcome of septic patients [5].

The heterogeneity of human patients with sepsis together with difficulties in controlling study conditions makes it complicated to work directly from human samples [8]. Murine models have been developed in an effort to create reproducible and rapid systems for studying sepsis pathogenesis [9]. Mice have similar genetic profiles to humans, and mouse models can be better controlled in terms of kinetics and interindividual variability [8]. Mouse models of sepsis have been commonly developed in order to study the evolution of the disease pathology and underlying mechanisms [[9], [10], [11]]. In some cases, mouse models of sepsis have been incorporated into the development pathway for novel therapeutic interventions [10,11], although most mouse models do not compare with human sepsis and fail to demonstrate clinical efficacy [8,[11], [12], [13], [14], [15]].

This review aimed to appraise those studies which utilized mouse models of sepsis to investigate biomarkers for sepsis prognosis prior to clinical studies. We sought to answer the following questions. What types of septic mouse models have been used and how closely do they reflect human sepsis? What technologies have been applied in discovering prognostic biomarkers using mouse models of sepsis? What prognostic biomarkers achieve the highest sensitivity and specificity and how clinically relevant are these biomarkers? We aimed to comprehend how mouse models were used to search for novel prognostic biomarkers of sepsis in order to optimize their use for future biomarker discovery.

## 2 Methods

**Fig. 1:**
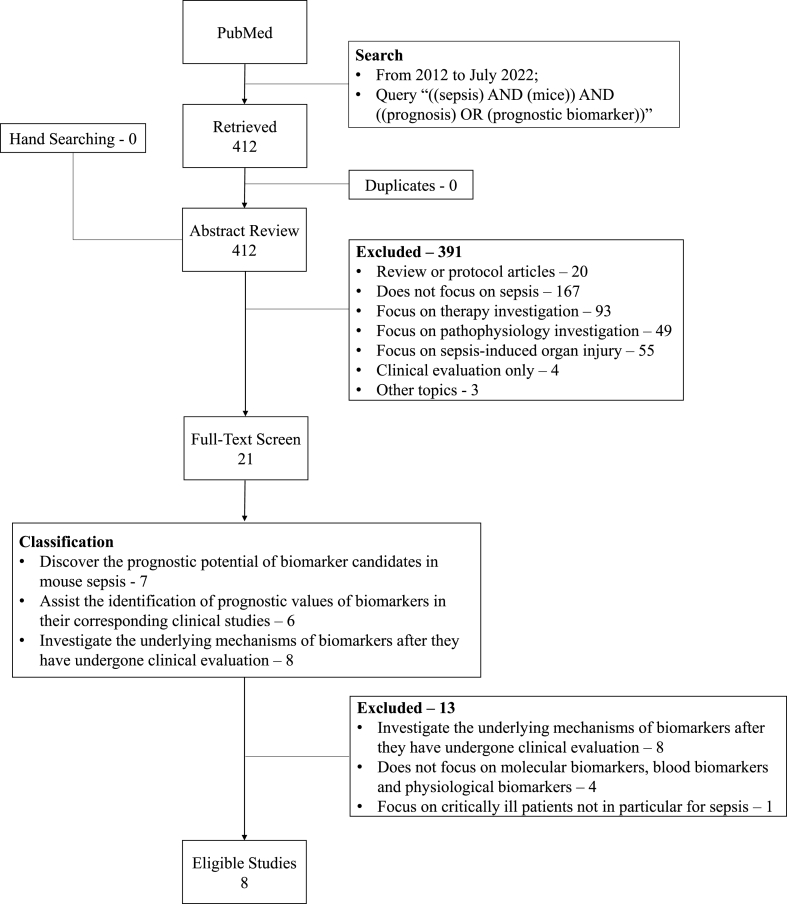
The schematic diagram of the literature review process.

We searched the PubMed database from 2012 to July 2022 using the query “((sepsis) AND (mice)) AND ((prognosis) OR (prognostic biomarker))” and retrieved 412 publications. We removed non-research articles, e.g., review articles. We excluded those publications not focusing on sepsis, sepsis prognosis and/or septic mouse models. We included the studies in which mouse models of sepsis were applied to discover prognostic potential of biomarker candidates directly in mice and/or assist the identification of prognostic value for human sepsis. We finally narrowed to eight relevant articles (Fig. 1). The full list of searched references with their research focuses is shown in supplementary file 1, Table S1.

We described the types of septic mouse models which have been utilized in the identified studies, compared and contrasted these mouse models with human sepsis in latter sessions, with reference to their strengths and weaknesses. We recorded the technologies applied on septic mouse models in order to discover prognostic values of biomarker candidates. For those biomarkers with clinical evaluations, we recorded the details of the clinical trials including the types of clinical study, the study population and the number of participants, and we presented the results of the survival analysis where this technique was applied to measure sensitivity and specificity of biomarkers.

## 3 Results

**Fig. 2:**
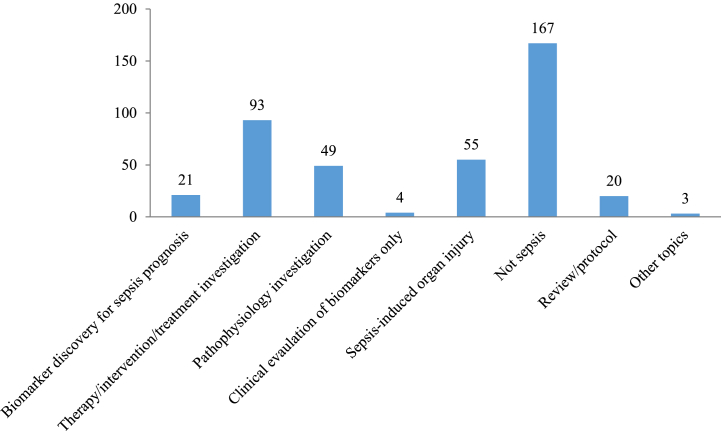
Research focuses of the publications which have been identified using the query of “((sepsis) AND (mice)) AND ((prognosis) OR (prognostic biomarker))” in PubMed from 2012 to July 2022. Y axis represents the number of articles.

**Fig. 3:**
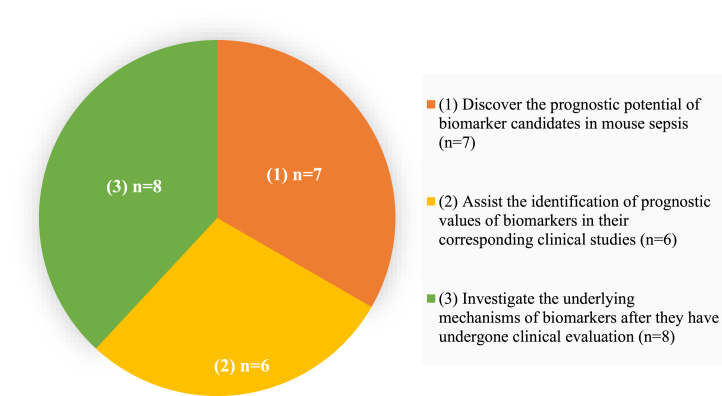
The application of mouse models of sepsis in the identified 21 publications with the topic of biomarker discovery for sepsis prognosis.

Among the retrieved 412 publications, 167 articles did not focus on sepsis; 93 addressed therapy/intervention/treatment investigation; 49 were about pathophysiology investigation; 55 were on sepsis-induced organ (e.g., kidney) injury; 20 were review or protocol articles; 4 were clinical evaluations without using septic mouse models; and only 21 were found to have used septic mice to discover biomarkers for sepsis prognosis (Fig. 2). Out of the 21 articles, 8 used mouse models of sepsis to investigate the underlying mechanisms of biomarkers after they have undergone clinical evaluation; 7 articles discovered the prognostic potential of biomarker candidates in mouse sepsis; and 6 used septic mouse models to assist the identification of prognostic values of biomarkers in their corresponding clinical studies (Fig. 3).

### 3.1 Biomarker discovery in mouse sepsis

Seven studies were found to apply mouse sepsis to demonstrate prognostic potential of candidates directly in septic mice. There is yet no following clinical assessment to support the findings. One study tested the potential of using peritoneal wash contents to predict mortality in septic mice, in which peritoneal wash contents were used as a source to measure immune parameters, e.g., IL-6 levels [16]; one study developed early label-free analysis of mitochondrial redox states by Raman spectroscopy to predict septic outcomes in mice [17]; and one study determined premorbid behavioural phenotype and acute-phase sickness behaviour to correlate with the mortality of septic old and adult male mice [18]. Focusing on molecular biomarkers and physiological biomarkers in this review, we did not include these three studies for appraisal in detail. The rest four studies identified the expression of six genes (CD247, DOCK2, IFI35, ITK, LCK, MED25) in peripheral blood, B7–H1 expression on neutrophils in peripheral blood and two physiological parameters, i.e., cardiac output at 2 h post-CLP and quantification of microcirculatory blood flow, to exhibit prognostic potential in mouse sepsis [[19], [20], [21], [22]]. Mimicking the traditional strategy of evaluating prognostic biomarkers in clinical studies, the former three studies all used mice to set survival and non-survival groups to do the comparison. Various techniques have been applied to discover biomarkers in mouse sepsis, representatively including screening differentially expressed genes (DEGs), measuring circulating levels of biomarker candidates and measuring physical parameters during sepsis progression. The next section will briefly describe each of these biomarkers with particular reference to their use in mouse sepsis and the corresponding discovery technology.

### 3.2 CD247, DOCK2, IFI35, ITK, LCK & MED25 gene expression in peripheral blood

The prognostic values of the expression of six genes (CD247, DOCK2, IFI35, ITK, LCK & MED25) in peripheral blood were identified using septic mouse models and RNA sequencing technology [19]. Survivor and non-survivor groups were produced by injecting low-dose of LPS (*Escherichia coli* lipopolysaccharide; 20 mg/kg) and high-dose of LPS (40 mg/kg) intraperitoneally into 8–12 weeks old male Kunming mice, respectively [19]. The peripheral blood of mice were sampled and screened for RNA extraction and differentially expressed genes (DEGs) between sepsis survival and non-survival mice [19]. Subsequently, gene ontology enrichment, protein–protein interaction (PPI) network and weighted gene co-expression network analysis (WGCNA) were used to analyse DEGs and recognize potential core genes [19]. Though there are no clinical cohort studies, the authors explored the available online database of human sepsis GSE65682 to plot survival curves of the core genes recognized in the mouse study. GSE65682 included the survival data of 479 septic patients (28 days) and their related peripheral blood cell gene data [19,23]. Overall, through mouse study and online patient data analysis, five genes (CD247, DOCK2, IFI35, ITK, and LCK) were reported to positively correlate with sepsis prognosis whilst only the expression of MED25 displayed a negative correlation [19].

### 3.3 B7–H1^+^ neutrophils in peripheral blood

B7–H1 is the primary ligand of Programmed cell death 1 protein (PD-1) [22,24]. Widely expressed on immune cells, PD-1 is involved in regulating a broad spectrum of immune responses [[25], [26], [27], [28]]. A high expression of PD-1 on peritoneal macrophages was detected in septic mice. In addition, PD-1 genetic polymorphism rs11568821 has been reported for its prognostic relevance in septic patients [29]. In this study, B7–H1 was first investigated for its pathogenic role in sepsis, for which mouse models of CLP-induced sepsis were used [22]. CLP models were established on 11–13 weeks old male C57BL/6J mice and B7–H1 gene deficient mice [22]. In this model, B7–H1 expression on various immune cells was upregulated during sepsis; B7–H1 deficiency protected mice from sepsis-induced death, lessened mouse organ damage and lowered inflammatory response; but B7–H1 deficiency showed no effect on changing the bacterial burden though it promotes more macrophages and neutrophils to migrate to the infectious site of peritoneum during sepsis [22]. B7–H1 deficiency and PD-1 deficiency were also compared to have similar effect of partially reversing the macrophages’ production of IL-6 and IL-10 ex vivo during sepsis; but unlike PD-1 deficiency, B7–H1 deficiency was shown unable to reverse the macrophage phagocytic function; and B7–H1 deficiency was shown no effect on the high expression of PD-1 on peritoneal macrophages and peripheral monocytes during sepsis [22]. Neutrophils were observed to have a distinct, polarized expression pattern of B7–H1 from other peripheral B7–H1^+^ cells [22]. Then, B7–H1^+^ neutrophils in peripheral blood (24 h after CLP) were compared to have a higher percentage in sepsis non-survival mice than survival mice (14-day mortality), indicating the B7–H1^+^ neutrophils in peripheral blood have the potential to be used as a prognostic biomarker in sepsis. Moreover, higher levels of inflammatory cytokines in blood correlated with the higher expression of B7–H1 on peripheral neutrophils [22].

### 3.4 Cardiac output at 2 h post-CLP

Cardiac function was used as a prognostic biomarker for sepsis in mice. Septic models, both of LPS injection (*E. coli* LPS; intraperitoneal; 5 mg/kg) and CLP, were constructed on 7–12 weeks old male C57BL/6 mice, and subsequent assessments were conducted at 2, 4, 6, 12 and 24 h after inducing sepsis [20]. Using both models, inflammatory cytokines including IL-1α, IL-1β, IL-6, TNF-α and IL-10, and a biomarker of cardiac stress, B-type natriuretic peptide (BNP), were elevated in plasma [20]. Speckle-tracking echocardiography, invasive hemodynamics, pulse-wave doppler and tissue doppler demonstrated consistent signs of systolic and diastolic dysfunction, suppressed cardiac output and myocardial strain [20]. In addition, blood pressure, body weight and surface temperature were reduced in both models [20]. A reduced cardiac output at 2 h after CLP at a cut-off value of <0.2279 ml/min could predict 48 h-mortality with 100% sensitivity and 85.5% specificity [20]. The assessment of cardiac function revealed different pathophysiological changes between LPS injection and CLP models; LPS caused only systolic dysfunction while CLP led to both systolic dysfunction and reduced preload [20]. Besides predicting mortality, measuring myocardial strain and cardiac output could also be used to monitor mouse recovery from sepsis after antibiotic treatment [20].

### 3.5 Quantification of microcirculatory blood flow

In order to demonstrate its prognostic value in murine sepsis, laser speckle contrast imaging was used to quantify mesenteric blood flow. Both LPS injection (*Salmonella typhimurium* LPS; intravenous; 12.5 mg/kg) and CLP models were used, and both models were constructed on male C57BL/6 mice (Table 1). Septic mice initially (within 9 h) exhibited compromised cardiac function and then (9 h–24 h) stabilized, as revealed by the measurement of systemic hemodynamics and echocardiography [21]. However, mesenteric blood flow quantified by the laser speckle contrast imaging exhibited a continuous decline over 24 h in both models [21]. Meanwhile, persistent metabolic acidosis and increased levels of creatinine and urea suggested that organ dysfunction also lasted over 24 h, mirroring the declining trend of mesenteric blood flow [21]. Hence, mesenteric blood flow was shown to be a more sensitive indicator of sepsis progression than hemodynamics and cardiac function in mouse models of sepsis. Fluid resuscitation which is commonly used to improve septic patient outcomes reversed the perturbed microvascular function/blood flow [21], suggesting that the quantification of microcirculatory blood flow could be used as an indicator of patient recovery from sepsis after treatment. The quantification of mesenteric blood flow may provide a sensitive biomarker for sepsis prognostication to assist in monitoring sepsis progression after applying interventions so as to guide the identification of novel, potential interventions to treat sepsis [21].

**Table 1:** Septic mouse models used to discover prognostic biomarkers of sepsis identified in the current literature review.

Biomarker	Septic mouse model	Mouse strain	Mouse age (weeks)	Mouse sex	CLP needle size (Gauge)	CLP number of punctures	LPS route	LPS dose (mg/kg)	Ref.
**Molecular marker**									
CD247, DOCK2, IFI35, ITK, LCK & MED25 gene expression in peripheral blood	LPS	Kunming	8–12	Male	–	–	I.P.	20 & 40	[19,30]
B7–H1^+^ neutrophils in peripheral blood	CLP	C57BL/6J	11–13	Male	22	Twice	–	–	[22,31]
Serum ApoA5	LPS	–	–	–	–	–	–	–	[25,32]
Plasma HSPA12B	CLP	C57BL/6J	6–8	–	22	Once & twice	–	–	[33,34]
Serum TREM-1	Bacteria (S*treptococcus progenies*)	C3H/HeN	–	Female	–	–	–	–	[19,35]
Serum miR-223	LPS & CLP	C57BL/6	6–8	Male	20	Twice	I.P.	2.5	[31]
**Physiological marker**									
Cardiac output at 2 h post-CLP	LPS & CLP	C57BL/6	7–12	Male	19	Once & twice	I.P.	5	[20]
Quantification of microcirculatory blood flow	LPS & CLP	C57BL/6	–	Male	19	Once	I.V.	12.5	[21]

Note: ApoA5 = Apolipoprotein A-V, HSPA12B = Plasma heat shock protein A12B, TREM-1 = Triggering receptor expressed on myeloid cells-1, - = Not applicable, -- = Not mentioned, LPS = Lipopolysaccharide; CLP = Caecal ligation and puncture, I.P. = Intraperitoneal, I.V. = Intravenous.

### 3.6 Assisting the biomarker discovery for human sepsis

Most mouse models do not compare well with human sepsis and discoveries later fail to demonstrate clinical efficacy [8,12,36]. It is generally accepted that the results obtained with the animal model are not always transposable to humans [8]. However, animal models are usually necessary in order to better understand mechanisms of disease, to confirm biological plausibility and to evaluate temporal relationships. Ultimately, clinical validation is necessary to confirm the translatability of new biomarkers after prior discovery in animal models.

The six identified biomarkers serum apolipoprotein A-V (ApoA5), serum miR-223, plasma heat shock protein A12B (HSPA12B), redox state of pentraxin 3 (PTX3), serum miR-133a and serum triggering receptor expressed on myeloid cells-1 (TREM-1) were initially studied in septic mice with the aim to support the subsequent clinical evaluation of their prognostic values in patients with sepsis. Apart from serum miR-223 which was shown to be ineffective, all the other five biomarkers demonstrated convincing prognostic potential in clinical studies.

As we will focus on circulating/blood biomarkers in this section, we will not discuss the redox state of PTX3. MiR-133a was investigated its prognostic value for critically ill patients but not in particular for sepsis, thus we will also not discuss it. The other four biomarkers will be briefly described for their nature and the discovery techniques used. Although the four biomarkers have been evaluated in clinical settings, only two, ApoA5 and HSPA12B, have reports on their clinical performance with a sensitivity and specificity (see Table 2).

**Table 2:** Clinical performance of prognostic biomarkers of sepsis identified in the current literature review.

Biomarker	Mouse sepsis	Human sepsis									
		Non-survivors (IQR)	Survivors (IQR)	*P* value	Cohort number	AUC (95% CI)	Sensitivity	Specificity	Cut-off point	Outcome	Ref.
Serum ApoA5 (Paediatric patients with sepsis)	Significantly increased at 24 h in LPS mice	793.7 (583.4–962.5) ng/mL	1219.4 (957.7–1938.6) ng/mL	0.009	101	0.789 (0.593–0.984)	75%	83.6%	822 ng/mL	Paediatric ICU mortality	[32]
Plasma HSPA12B (Severe sepsis)	Significantly elevated at 6 h and peaked at 24 h in CLP mice	2.9 (1.4–6.7) ng/mL	0.9 (0.5–1.3) ng/mL	<0.001	66	0.782 (0.654–0.909)	76.9%	82.5%	1.466 ng/mL	28-day mortality	[33]

Note: ApoA5 = Apolipoprotein A-V, HSPA12B = Plasma heat shock protein A12B, LPS = Lipopolysaccharide, CLP = Caecal ligation and puncture; IQR = Inter-quartile range, AUC = Area under the receiver operating characteristic (ROC) curve, 95% CI = 95% confidence interval, ICU = Intensive care unit.

### 3.7 Serum ApoA5

ApoA5 modulates triglyceride homeostasis, whilst elevated plasma triglycerides occur in the acute-phase of sepsis [37]. The clinical values of serum ApoA5 in paediatric patients with sepsis were investigated; LPS-treated mice were used to assist the investigation. Initially, a proteomic analysis focusing on the proteins involved in lipid metabolism was conducted to reveal the significant lifted levels of serum ApoA5 in septic mice [32]. However, little information of the septic mouse models, i.e., the administration route and dose of LPS, and the strain, age and sex of mice, were provided. Subsequently, ApoA5 was evaluated in paediatric patients with sepsis. A prospective study was conducted, recruiting 101 patients with sepsis (age 28 days to 14 years; diagnosis within 24 h of admission; 90 survivors) admitted to the paediatric intensive care unit (PICU). Serum ApoA5 concentration was measured by ELISA. The AUC of serum ApoA5 levels was 0.789. At a cut-off value of 822 ng/mL, the sensitivity was 75% and the specificity was 83.6% for predicting PICU mortality [32].

### 3.8 Plasma HSPA12B

HSPA12B, mainly located in endothelial cells and responsible for angiogenesis and endothelial functions [[33], [38], [39]]. HSP12B may present in circulating systems of patients with endothelial injury, and the clinical values of its presence in plasma of patients with severe sepsis were investigated. Plasma HSPA12B is elevated in a CLP mouse model (6–8 weeks old C57BL/6J, sex not specified) and correlates with disease severity, in which the IP puncture numbers, either once or twice, were used to produce the different disease severity [33]. Subsequently, in a prospective study on patients with severe sepsis from ICU (40 survivors and 26 non-survivors), ELISA was used to measure plasma HSPA12B levels. The AUC for predicting 28-day mortality in severe sepsis was 0.782. At a cut-off value of 1.466 ng/mL, the sensitivity was 76.9% and the specificity was 82.5% [33].

### 3.9 Serum TREM-1

TREM-1 is an immunoglobulin expressed on the surface of neutrophils and macrophages, which can amplify inflammatory responses [[40], [41], [42]]. The plasma levels of soluble TREM-1 (sTREM-1) are able to differentiate sepsis from systemic inflammatory response syndrome; within sepsis, survivors show lowered levels of sTREM-1 whereas non-survivors do not [43,44]. The prognostic value of TREM-1 in particular for *Streptococcus progenies*-induced sepsis was investigated in both mouse models and patients. The mouse model of *S. progenies* sepsis was constructed on C3H/HeN female mice by intravenously inoculating 10^5^ CFU of *S. progenies* strain A20 [35]*.* Using this mouse model, TREM-1 expression was upregulated in the liver and peritoneal cells during *S. progenies* infection; and the serum TREM-1 levels correlated positively with the bacterial loads in the blood and liver of *S. progenies* infected mice [35]. Therefore, the serum TREM-1 levels reflect the severity of *S. progenies* infection in mice. The results from mice were then translated into patients with streptococcal toxic shock (STSS; n = 18) [35]. Consistent with infected mice, STSS patients suffered higher levels of plasma TREM-1 which correlated significantly with the disease severity defined by SAPS II (Simplified Acute Physiology Score) [35]. In addition, 15 STSS survivors demonstrated declining levels of plasma TREM-1 over 72 h after infection, whereas 3 non-survivors either displayed extremely high levels or increased sharply of TREM-1 in plasma [35]. The clinical observation suggested the potential of using soluble TREM-1 in blood as a prognostic indicator for *S. progenie*-induced sepsis.

### 3.10 MicroRNA-223 serum levels

The identified study reported negative values of miR-223 for being used as a sepsis biomarker. Previous clinical studies have reported contradictory results of using miR-223 serum levels as a septic biomarker [[45], [46], [47]]. Initially, serum levels of miR-223 in septic mouse models were measured using quantitative real-time PCR (qPCR) [31]. Two types of septic models, LPS injection (intraperitoneal; 2.5 mg/kg) and CLP, were applied using 6–8 weeks old male C57BL/6 mice (Table 1). However, dissimilar changes in serum levels of miR-223 between these two models were found [31]. LPS injection induced elevated levels of miR-223 in mice, whereas CLP surgery did not show any effect on changing miR-223 levels [31]. This disaccord may reflect the innate differences between the two types of septic models [31]. LPS injection induces an acute sterile inflammation but CLP features bacteraemia with a delayed course of inflammation, which may indicate a role of miR-223 in differentiating aseptic inflammation from bacterial sepsis. Nevertheless, in this study, miR-223 serum levels did not appear to be useful as a circulating biomarker to indicate bacterial sepsis in mouse models [31]. MiR-223 was then evaluated for its clinical use in differentiating healthy controls from critically ill patients, diagnosing patients with or without sepsis and predicting prognosis of critically ill patients [31]. A well-characterized cohort study was executed, recruiting critically ill patients admitted to ICU [31]. The total cohort number was 296, including 221 ICU patients and 75 healthy volunteers. Of the ICU patients, 137 had sepsis, 84 no sepsis, 127 survived and 94 died. Of the non-survivors, 49 died in ICU and 45 died during a three-year follow-up. miR-223 in serum and tissue were measured with qPCR [31]. No significant differences in miR-223 serum levels were found between healthy volunteers and critically ill patients, between septic and non-septic patients, or between survivors and non-survivors [31]. Moreover, no correlations were found between miR-223 serum levels and classical biomarkers such as CRP, PCT and APACHE-II scores [31]. Thus, consistent with mouse experiments, this prospective study also demonstrated no evidence to support the use of miR-223 as a septic biomarker [31].

In the discovery of ApoA5 and miR-223, septic mice were mainly used to measure the biomarker levels and then confirm whether the changes are consistent with septic patients in following clinical trials. In the discovery of HSPA12B and TREM-1, besides measuring the changes of candidate biomarkers in mouse sepsis, the disease severity of mouse sepsis, either controlled by the CLP puncture numbers or monitored by bacterial loads, was used to analyse the correlation with candidate biomarkers. In terms of using septic models to assist the biomarker discovery for the prognosis of human sepsis, the techniques applied on mice seem to be limited.

### 3.11 Septic mouse models

Among the eight studies mentioned above, five studies used C57BL/6 mice, one study used Kunming mice and one used C3H/HeN mice to establish mouse sepsis (Table 1). C57BL/6 strain has been widely used for general purposes, which is also commonly used to establish sepsis models [48,49].

### 3.12 Genetic factor

C57BL/6 and C3H/HeN mice are inbred strains while Kunming mice are outbred strains. For inbred mice, their almost identical genetics facilitate the interpretation of experimental results and ensure the reproducibility; for outbred strains, due to their diverse genetics, mice are more robust and prolific. Generally, inbred mice are more widely used in sepsis research than outbred mice [50]. Controversial findings of mouse strains in response to experimental sepsis have been reported. For example, though discordant susceptibility to experimental fungal sepsis was observed between C57BL/6 and CD-1 mice [51], no significant difference in establishing LPS injection models was found between the inbred strain C57BL/6 and another widely used outbred strain ICR [52]. Yet, Radulovic referred that different mouse strains hold different susceptibility towards LPS [49]. In the current literature review, different doses of LPS were required to establish sepsis between C57BL/6 mice and Kunming mice. This is consistent with Stortz's review in which the differences between mouse strains are illustrated to affect the susceptibility to pathogens even among inbred strains. Genetic differences result in variations in the immune responses of different strains towards infections, e.g., C57BL/6 mice exhibiting a Th1-predominant response while Balb/c mice presenting a Th2-predominant response [50]. Therefore, though no limits and recommendations in choosing mouse strains have ever been stated formally, it is always helpful for researchers to consider the genetic background of their mice to optimize their strategy in establishing murine sepsis.

### 3.13 Sex

Five studies used male mice to produce sepsis, two studies did not mention the mouse sex, and only the *S. progenies*-induced model used female C3H/HeN mice (Table 1). Sex-based differences in susceptibility to sepsis in mice have already been reported [50,[52], [53], [54]]. Compared with female mice, male mice are more susceptible to infection leading to a higher death rate [52,54]. In terms of neutrophil recruitment and proinflammatory cytokine release, female mice displayed lower levels than male mice [[52], [54]]. Similar with the sex-based differences in septic mice, female patients clinically were also shown to be conferred with more protection against sepsis than male patients which may be explained by the protective effect of female hormones [[55], [56], [57]]. Therefore, there would be a sex factor creating noise to affect the observation of disease progressions if mixed sexes of mice were used. Choosing only male mice which could deliver greater responses under sepsis conditions appears to be more recommended for establishing septic models, which actually has become a predominant situation in experiments [50].

### 3.14 Age

With regards to the mouse age, five studies provided this information; the mice age ranged from 6 weeks to 13 weeks. In fact, the age-dependent differences have been recognized long before [50,52,53,[58], [59], [60]]. After exposure to LPS, adolescent mice (8 weeks old) demonstrated stronger vitality as reflected by the higher survival rates and longer latency to death than adult mice (13 weeks old) [52]. Middle-aged mice (36–40 weeks old) exhibited exaggerated microglial responses to LPS-induced systemic inflammation and expressed higher levels of TNF-a, IL-1a and IL-6 than young mice (8 weeks old) [58]. Aged mice (98–102 weeks old) have also been compared with young mice (6–7 weeks old) to reveal less tolerance to the LPS-induced lethality and generate significantly higher levels of proinflammatory cytokines (i.e., TNF-α, IL-1a and IL-6) [59]. In response to CLP, young mice (16 weeks old) acquired the lowest mortality than mature mice (48 weeks old) and aged mice (96 weeks old), and young mice also exhibited lower levels of IL-6 and TNF-α than aged mice [61]. Thus, in order to avoid those age-acquired alterations in survivability and systemic inflammation and meanwhile acquire mature inflammatory responses as well as reducing cost, young mice are more preferred to be used to establish septic mouse models [50,60]. Consistently choosing male gender and younger age may make experiments comparable and standardized. However, sepsis is largely a condition of elderly patients and it affects both male and female. Therefore, is it more appropriate to use mouse models with a balance of gender and older age?

### 3.15 Model type

In the eight articles, mouse models of sepsis generally fall into three widely reported categories: 1) alteration of the animal's endogenous protective barrier, e.g., CLP; 2) exogenous administration of a viable pathogen; or 3) exogenous administration of a toxin, e.g., LPS. Septic mouse models hold an irreplaceable position for us to acquire fundamental information related with the mechanism of sepsis pathophysiology and treatment. Different types of septic models come with their own strengths and weaknesses which have been reviewed extensively by numerous experts in the field [9,10,13,48,62], thus, have not been covered in detail here.

CLP and LPS injection were the models predominately used in the above eight appraised studies (Table 1). In two studies, B7–H1^+^ neutrophils and HSPA12B were discovered using the CLP model; in two studies, the expression of six genes (CD247, DOCK2, IFI35, ITK, LCK & MED25) and ApoA5 were discovered using the LPS injection model; and in three studies, miR-223, cardiac output at 2 h post-CLP and quantification of microcirculatory blood flow were identified using both CLP and LPS injection models. Dissimilar changes between these two models were found for miR-223, and clinical findings were eventually proven to be consistent with CLP models. This phenomenon may reflect the innate differences between the two types of septic models. LPS injection induces sterile inflammation yet CLP features bacteremia. Thus, it appears to be safer to test candidate biomarkers in multiple, different models prior to large-scale clinical trials [48]. In the next sections, we will review the CLP and LPS models.

CLP model involves anesthetizing mice and performing a midline laparotomy to expose, ligate and puncture the cecum [8,9,63]. Faecal materials then leak into the peritoneum to induce peritonitis. Enteric bacteria translocate into the bloodstream and finally activate systematic inflammation [8,9,63]. This septic peritonitis model has been considered as the gold-standard model of sepsis, particularly mimicking ruptured appendicitis or perforated diverticulitis. It is most useful for studying human sepsis progression from peritonitis [9,13,63]. In this model both proinflammatory and anti-inflammatory responses are activated, form early hyperdynamic and late hypodynamic phases and produce multiple organ failure and metabolic alterations, closely resembling human sepsis [9,[62], [63], [64]]. However, therapeutic interventions which have shown effective in this model mostly failed to achieve efficiency in humans, indicating that CLP model may not fully reproduce the complexity and intrinsic heterogeneity of human sepsis [13,63]. This model avoids the extra preparation and administration of toxins or pathogens, but induces polymicrobial sepsis directly using the diverse intestinal microbiota of the host itself through destroying the gut barrier [13,62]. Needles are used to puncture the cecum to impair the gut integrity. The size of needles and the numbers of punctures are used to manage the degree of disease severity [13,33]. Among the identified studies using CLP models, the needle size varied from 19-gauge to 22-gauge, and the puncture numbers varied from once to twice [[20], [21], [22],^33^,^31^]. Besides the variations in needle size and puncture numbers, the amount of cecum ligated, the uncontrolled load of leaking faecal materials, the differences in age, sex and strain of mice, the anaesthetic choice and the laparotomy technique within and between different studies make it difficult to standardize the CLP model establishment, actually resulting in poorly reproducible models [9,13,48,62].

The injection of LPS produces sterile inflammation in mice, inducing systemic inflammatory responses in the absence of an ongoing infection [62]. LPS injection is commonly via intraperitoneal (*I.P.*) route, and sometimes via intravenous (*I.V.*) route. Among the five identified studies using LPS injection models, three used the *I.P.* route, one used *I.V.* route and one did not provide this information [32]. The median lethal dose (LD_50_) of LPS in mice is about 1–25 mg/kg, which may be influenced by the age, sex and strain of mice. For C57BL/6 male mice used in the identified studies, 2.5 mg/kg and 5 mg/kg of *I.P.* LPS were administrated to 6–8 weeks old mice and 7–12 weeks old mice, respectively; and 12.5 mg/kg of *I.V.* LPS was also utilized but without mentioning the mouse age (Table 1). For Kunming male mice (8–12 weeks old), higher doses of *I.P.* LPS (20 mg/kg and 40 mg/kg) were required to produce sepsis with different severity (Table 1). LPS is a relatively pure chemical deriving from the outer membrane of a gram-negative bacterium, such that LPS can be measured reliably. The exact dose and the route of administration of LPS can be standardized, reducing the inter-animal variability and ensuring the experiments to be readily reproducible. Sepsis development/progression in mice could be regulated by changing the LPS amount [48,62], as an example of using different doses of LPS to set survival and death groups in the discovery of six genes (CD247, DOCK2, IFI35, ITK, LCK and MED25) [19]. However, there is a striking divergence in LPS sensitivity between mice and human beings. Human beings are more sensitive to the toxic effects of LPS than mice, with the typical dose inducing fever symptoms and the release of proinflammatory cytokines only being about 2–4 ng/kg [10]. Moreover, the disease progression in LPS models dramatically differs from human sepsis and other infection models. Apparently, LPS models lack the host-pathogen interactions. On the one hand, it takes hours to days to initiate sepsis and progress to multiple organ failure in mouse models, but this progression occurs over days to weeks in human patients [9]. On the other hand, injection of LPS in mice induces a strong, short-term increase of plasma proinflammatory cytokines (e.g., TNF-α, IL-1β and IL-6), in comparison to the lower and progressive cytokine increase in human sepsis [[13], [14], [15],^48^]. These remarkable divergences imply that data obtained using LPS models may not work for the human cases [10]. This endotoxemia model does not accurately portray human sepsis.

## 4 Discussion

This review appraised eight studies reporting potential prognostic biomarkers in mouse models of sepsis, four of which advanced to clinical studies. The search strategy is focused. We sought to understand how mouse models were used to search for novel prognostic biomarkers in sepsis. The knowledge and perspectives we formed will lead us to know what may be achieved with our own early attempts and come up with lessons which may guide the use of septic mouse models for future discovery.

This literature search only identified eight publications using mouse models with aims to discover biomarkers for sepsis prognosis, which is small. This phenomenon reflects the fact that, although mouse models of sepsis have been commonly developed as proxy for the human condition to study sepsis, most of them are used to investigate the pathophysiology and evaluate novel therapeutic interventions with their pharmacokinetics, toxicity and underlying mechanisms [[9], [10], [11],^13^]. These studies on intervention or pathophysiology might provide information on possible biomarkers. However, the focus of our paper is to understand how other researchers used mouse models to discover novel prognostic biomarkers of sepsis. Hence, we have excluded these studies on intervention or pathophysiology from being discussed in our review. Nonetheless, we believe that there is much valuable information in these excluded articles. Therefore, we have organised the information of all 412 articles in the supplementary file and categorised them according to their research focuses. We believe this information will help other researchers quickly retrieve their interested biomarkers-related articles. In addition, any possible biomarkers could be explored in a human sepsis database, such as the GSE65682 (www.ncbi.nlm.nih.gov/geo/query/acc.cgi?acc=GSE65682), for correlation. In our future study on biomarker discovery, we would like to incorporate the exploration of potential biomarkers in this type of human dataset.

As mentioned, the discovery techniques applied on mice are also very limited, in which case mouse sepsis was mainly used to detect the changes of biomarker levels during sepsis. Mice have been used to set survival and nonsurvivable groups, and candidate biomarkers were then compared between the two groups, mimicking the traditional strategy of evaluating prognostic biomarkers in clinical studies. Other than that, it is hard to categorize new strategies of biomarker discovery from this small number of studies. Screening DEGs in mice after setting survival and nonsurvivable groups may be novel but even this number of studies are very few. These limitations indicate a huge space of further exploring how to use septic mice to discover prognostic biomarkers for sepsis. Mice will remain promising for biomarker discovery but it requires thorough development in both model itself mimicking human sepsis and techniques as well as discovery strategy. We have formed three perspectives to fill in this space, which will be discussed below.

Firstly, it is important to optimize current models to maximize the extent to mimic human sepsis. The discovery mostly relies on human samples, and this lack of using mouse models may be due to the differences between human sepsis and mouse sepsis. As mentioned before, most mouse models poorly reflect human sepsis and fail to demonstrate clinical efficacy [8,[11], [12], [13], [14], [15]]. Knowledge of clinical situation and current mouse models of sepsis will guide improvements for the future and the discovery of better biomarkers. For instance, most cases of sepsis are due to community acquired respiratory infection invading the lung. However, as for the commonly used LPS model, the predominant route of toxin administration is through intraperitoneal injection which may provide a good model for abdominal sepsis but may not reflect disease progression in sepsis due to respiratory causes. Therefore, respiratory route, e.g., intranasal instillation, of administrating LPS shall be developed to establish a model of respiratory sepsis rather than abdominal sepsis.

Secondly, it may hold advantages to test biomarkers in multiple, different models prior to large-scale clinical trials, as inspired by the contrasting results of testing miR-223 levels between LPS injection and CLP. Most studies used only one type of septic models to test biomarkers. However, different models come with their own strengths and weaknesses which have been reviewed numerously and also briefly described in the section of “Septic mouse models”. For instance, CLP features a delayed course of inflammation but LPS injection induces an acute sterile inflammation. The innate differences between different types of septic models may lead to striking differences in biomarker changes. Though testing biomarkers in multiple models may increase preclinical labour and cost, it could offer stronger evidence to support clinical evaluation and lower the risk of clinical failure. On the other hand, new U.S. Food and Drug Administration (FDA) regulations aim to minimise the use of pre-clinical models [65]. Therefore, it remains essential to choose the most proper model after weighing with the measured parameters and the overall objectives of each study to ensure study aims getting addressed to the greatest extent [66]. As mentioned, each of the current models has its own strengths and weaknesses, which prevents any single one from being the perfect one [9]. Up to the experimental situation, the most proper model should be prioritised to be used, and if condition permits, multiple, different models are also suggested to be tested in order to provide stronger evidence to support clinical evaluation and lower the risk of future clinical failure.

Thirdly, advanced techniques can be applied in mice [8]. Mice are easily manipulated and standardized. Dealing with human samples is complex, which may involve severe ethical issues limiting the access to various technologies. Similar with the application of screening DEGs in mice, cell-free DNA, proteomics, metabolomics and lipidomics could also be applied in septic mice to discover biomarkers. Identified candidates could then be translated into human sepsis. Mice have strong homology with humans, are well studied, and can be used to control the kinetics of the development of the pathology and to standardize experimentation, facilitating the identification of potential biomarkers. Mouse models remain promising to play an essential role in the discovery of new biomarkers.

Half of the identified biomarkers advanced to clinical evaluation, but only ApoA5 and HSPA12B provided data from survival analysis. Though both achieved similar AUC, sensitivity and specificity, ApoA5 focuses on ICU mortality of paediatric patients, and HSPA12B focuses on 28-day mortality of patients with severe sepsis. Also, there is no common standard used during their clinical evaluation, making it difficult to compare their clinical performance. Hence, biomarker evaluation ought to not only require researchers to provide the data of prediction performance, i.e., AUC, sensitivity and specificity, but also use some common standards, such as APACHE II score and CRP, to report results, which will facilitate the comparison between identified biomarkers so as to aid researchers to prioritize potential biomarkers for further advance.

None of the clinical trials features a large cohort number more than 300, and none of the biomarkers have been evaluated in repeated, large studies (Table 2). Although the reduced cardiac output at 2 h post-CLP could achieve 100% sensitivity and 85.5% specificity to predict the 48 h-mortality for septic mice, this has not been validated in human cases. The biomarkers, ApoA5 and HSPA12B, which were the only two biomarkers reported their clinical performance, demonstrated consistent significant changes in mouse sepsis with human sepsis. However, the AUCs were below 0.8, the sensitivities were 75%–77%, and the specificities were 83%–84% in clinical evaluations (Table 2). Thus far the research community has not identified any biomarker or biomarker platforms that achieve sufficiently high sensitivity and specificity for use in clinical practice. The studies in this review are recent (between 2012 and 2022) and suggest that current mouse models are insufficiently similar to human sepsis for biomarker discovery for sepsis prognosis.

## 5 Conclusions

Though mouse models have been widely used in sepsis research, the number of studies and strategies using septic mice to discover prognostic biomarkers of sepsis are very limited, and the identified biomarkers still require substantial assessment of their clinical potential. Current mouse models of sepsis cannot truly reflect human sepsis and need to be further optimized to better conform to human sepsis. Nonetheless, mouse models can create reproducible and rapid sepsis, hold the capability to access various techniques and will remain promising to facilitate the discovery of biomarker platforms with sufficient sensitivity and specificity for use in clinical practice.

## Authors contributions

YJ conceived the study, performed the literature search, drafted the manuscript, and approved the submitted version of the manuscript. CT revised the manuscript for critical content, and approved the submitted version of the manuscript. TR conceived the study, revised the manuscript for critical content, and approved the submitted version of the manuscript.

## Funding

This research did not receive any specific grant from funding agencies in the public, commercial, or not-for-profit sectors.

## Declaration of competing interest

The authors declare that they have no known competing financial interests or personal relationships that could have appeared to influence the work reported in this paper.
